# Personalized Dosimetry for Radionuclide Therapy Using Molecular Imaging Tools

**DOI:** 10.3390/biomedicines4040025

**Published:** 2016-11-15

**Authors:** Michael Ljungberg, Katarina Sjögreen Gleisner

**Affiliations:** Department of Medical Radiation Physics, Lund University, 221 85 Lund, Sweden; katarina.sjogreen_gleisner@med.lu.se

**Keywords:** dosimetry, radionuclide therapy, SPECT, reconstruction, quantification, Monte Carlo

## Abstract

For treatment of systemic malignancies, when external radiation therapy is not applicable, radionuclide therapy can be an alternative. In this form of therapy, radionuclides are administered to the patient, often in a form where the radionuclide is labelled to a molecule that plays the active part in the localization of the tumor. Since the aim is to impart lethal damage to tumor cells while maintaining possible side-effects to normal tissues at tolerable levels, a proper and accurate personalized dosimetry should be a pre-requisite. In radionuclide therapy, there is a need to measure the distribution of the radiopharmaceutical in vivo, as well as its re-distribution over time, in order estimate the total energy released in radioactive decays and subsequent charged-particle interactions, governing the absorbed dose to different organs and tumors. Measurements are usually performed by molecular imaging, more specifically planar and SPECT (Single-Photon Emission Computed Tomography) imaging, combined with CT. This review describes the different parts in the dosimetry chain of radionuclide therapy. Emphasis is given to molecular imaging tools and the requirements for determining absorbed doses from quantitative planar and SPECT images. As example solutions to the different problems that need to be addressed in such a dosimetric chain, we describe our tool, Lundadose, which is a set of methods that we have developed for personalized dosimetry.

## 1. Introduction

In cancer treatments using radiation, it is generally accepted that it is the absorbed dose, i.e., the deposited radiation energy per unit mass, which is the fundamental quantity for coupling to the radiobiological effect. Formally, the absorbed dose, *D*, is defined as the mean energy imparted, $d\bar{\varepsilon }$, to the matter in a volume with mass d*m*, according to:

(1) $$D=\;\frac{d\bar{\varepsilon }}{dm}$$

In principle, the absorbed dose is defined in a point, and its value can thus vary between different parts of an organ or tissue [1]. In nuclear medicine, such heterogeneities are evident when looking at a small scale, and quoted absorbed dose values generally refer to the mean absorbed dose, as formally defined:

(2) $${\bar{D}}_{T}=\frac{1}{m_{T}}\int_{m_{T}}D\;dm$$

where $m_{T}$ is then the mass of a tissue, or organ, or some other target volume for which the absorbed dose is determined [1].

Internal dosimetry, that is dosimetry for internally-distributed radionuclides, generally relies on descriptions of the radiation-energy transport from a source to a target region, as described by the following equation:

(3) $${\bar{D}}_{T}=\int_{t_{1}}^{t_{2}}A\left(r_{S},t\right)\;dt\cdot \frac{1}{m_{T}}\underset{i}{\sum }n_{i}E_{i}\cdot \phi \left(r_{T}\leftarrow r_{S},E_{i}\right),$$

where the integral describes the total number of decays occurring in the source region during the time interval *t*_2_ − *t*_1_ (also called the time-integrated activity). The summation describes the fraction of the kinetic energy *E* released by each particle type, *i*, with *n* emitted particles per radioactive decay, which will be absorbed in the target volume. In principle, the equation is valid for different kinds of volumes, from organs down to cells, but for smaller volumes, the stochastic nature of radiation interaction makes the mean value less representative of the actual energy deposition. The product of the summation term and the inverse of the target mass in Equation (3) is often called the S value. For risk estimates in diagnostic nuclear medicine examinations, the amount of energy that is transported from the source to the target is often estimated based on computer models representative of a population, but for internal dosimetry in cancer therapy, the terms in Equation (3) need to be estimated on an individual basis.

Personalized dosimetry based on molecular imaging is a field that probably will become more important in the near future. In the new EU directive 2013/59/EURATOM Article 56, it is stated regarding optimization that “For all medical exposure of patients for radiotherapeutic purposes, exposures of target volumes shall be individually planned and their delivery appropriately verified taking into account that doses to non-target volumes and tissues shall be as low as reasonably achievable and consistent with the intended radiotherapeutic purpose of the exposure”. In Chapter II, Definitions, it is further stated that “’radiotherapeutic’ means pertaining to radiotherapy, including nuclear medicine for therapeutic purposes”.

At our institution, we have been working with the development of dosimetric methods for radionuclide therapy for many years and have compiled these methods into a package that we call the Lundadose program. Most of the software is written in the programming language IDL (Harris Geospatial Solutions), and it is a complete stand-alone program. This has the advantage of being vendor independent in the development of molecular imaging tools. All image data are exported to Lundadose through Dicom files. Although the program is currently not available for general use, this article describes the most important methods and procedures and, as such, should be viewed as examples of how different problems encountered in radionuclide-therapy dosimetry can be solved. Thus, the overall aim of this review is to highlight the different steps that need to be taken in order to resolve the complexity and to accomplish personalized dosimetry in radionuclide therapy.

## 2. Comparison between Radionuclide Therapy and External Beam Radiotherapy

When comparing External Beam Radiation Therapy (EBRT), where the source of radiation is generally a linear accelerator, with radionuclide therapy, where the radiation source is an internally-administered radionuclide, there are striking similarities, but also fundamental differences in the way the absorbed doses are delivered and calculated. Both therapy modalities are based on energy depositions by charged particles (mainly electrons, but also protons and α particles). In EBRT, the electron fluence is generated by Compton interactions between the photons in the beam and electrons in tissues, while for RNT, the electrons are released in radioactive decays (often β-particles). In both modalities, the electrons lose their kinetic energy by subsequent interactions with molecular electrons in tissues. However, the geometry of the radiation source is in EBRT well defined, and the irradiation can be turned on and off. The shape of the radiation beam can be dynamically adjusted by multi-leaf collimators for different irradiation angles, thus optimizing the tumor absorbed-dose distribution while sparing normal tissues. The absorbed-dose rate is generally high, and the radiation delivery is accomplished during a couple of minutes. As long as the electron density of irradiated tissues is uniform, the energy deposition along the beam can be regarded as relatively uniformly distributed. RNT, on the other hand, relies on the accumulation of radiopharmaceuticals in target tissues. The accumulation in tumors and normal organs depends on the physiological mechanisms that govern the distribution of the radiopharmaceutical in the body, as well as cellular properties, such as the expression and turnover of receptors. The irradiation intensity, i.e., the absorbed-dose rate, thus changes in time. To a first approximation, it can be seen as exponentially decreasing with a rate that is governed both by the radionuclide physical half-life and the biological half-life of the radiopharmaceutical. The absorbed-dose rate is generally significantly lower than in EBRT, and irradiation extends over a long time (days). The pattern of radiopharmaceutical accumulation, when looking at a small scale, is almost always heterogeneous. Thus, the absorbed-dose distribution is in many cases heterogeneous, where the degree of heterogeneity is governed by the range of the charged particles that are emitted at decay. Radionuclides used often emit charged particles with different energies, including also low-energetic Auger-electrons that give rise to a locally, very high absorbed doses.

## 3. The Dosimetry Chain for Radionuclide Therapy (RNT)

**Figure 1:**
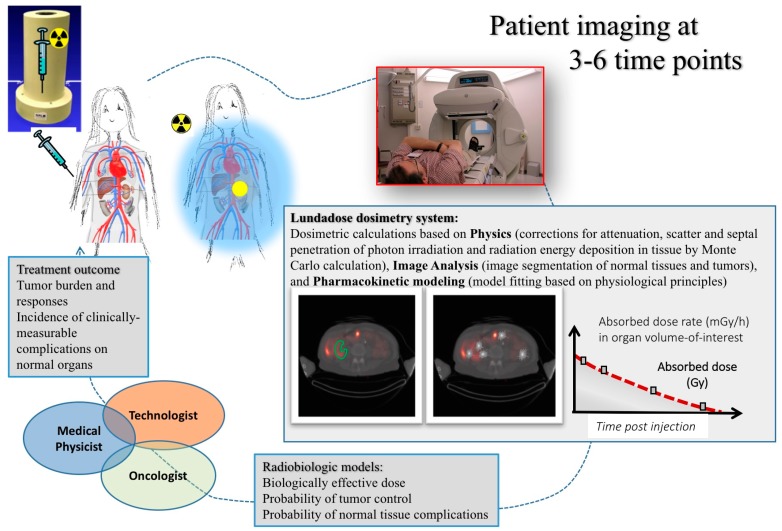
Overview of the chain for personal dosimetry using molecular imaging.

Figure 1 shows an overview of the dosimetry chain for RNT. Successful implementation relies on a close collaboration between oncologists, medical physicists and technologists. The dosimetry process can be grouped into three major parts. The first part deals with the physics that needs to be addressed in quantitative imaging and image-based dosimetry, including appropriate corrections for photon attenuation, scatter, camera limitations, as well as voxel-based calculation of the distribution of energy depositions. The second part relates to image analysis, including image registration, image segmentation and classification of normal tissue structures and tumors in the images. Thirdly, pharmacokinetic modelling needs to be applied to data obtained from images acquired at different time-points, in order to calculate the total amount of deposited radiation energy during the whole course of treatment, i.e., the absorbed dose. In addition to the physical quantity of the absorbed dose, other radiobiology-based parameters, such as the Biologically-Effective Dose (BED) and the Normal-Tissue Control Probability (NTCP), may be considered. The overall aim is to be able to predict an activity to be administered to an individual patient, in order to give a treatment that is considered optimal in terms of an adequate tumor control while minimizing potential side-effects to normal tissues.

The evidence base for the use of dosimetry in the clinical setting has been nicely summarized in an article from 2014 [2]. For instance, Barone et al. [3] found that kidney toxicity induced by ^90^Y peptide receptor RNT, quantified as the creatinine clearance loss per year, correlated well with renal BED. In MIRD Pamphlet No. 20 [4], these data were combined with data from external-beam radiotherapy to find that the incidences of renal complications were comparable for the same BED. Both Strigari et al. [5] and Chiesa et al. [6] described relationships between the normal tissue complication probability and the liver absorbed dose or BED received during ^90^Y microsphere treatments.

## 4. Scintillation Camera Imaging

The scintillation camera is the most common imaging system used in radionuclide therapy. The basic camera principle is that photons, emitted from and transmitted through the patient, generate a signal only if passing through a collimator and being detected by a scintillation detector. The deposited energy by the photons is here converted to visible light photons that subsequently are collected and amplified collectively by many Photon-Multiplication Tubes (PMTs). The centroid of the detected signals from all PMTs weighted by their position is then used to determine the position for a particular interaction in the crystal, and this finally forms the content in the image. The collimator is constructed as a lead sheet with thousands of parallel holes. The idea is here that the collimator discriminates against photons that travel in directions that are not approximately parallel to the axis of the collimator hole. Because of the collimator construction, optimized for the particular photon energy, the spatial resolution is in the order of 8–15 mm. This means that the distribution of the radioactive source in the patient is generally not possible to resolve on a small-scale level.

Ideally, when planning of a therapy with radionuclides, one should first make a dose planning of the treatment by, as a first step, determining the biokinetics of the radiopharmaceutical. Planning can be achieved by a tracer amount followed by sequential scintillation camera imaging. However, the scintillation camera used for this purpose has several inherent limitations, discussed below, that restrict the spatial resolution of the images and the accuracy in the determination of the time-integrated activity.

### 4.1. Planar Imaging

In planar imaging, the final images are two-dimensional projections. This means that the signal at a specific location in the image is built up from photons emitted along projection lines. Thus, if one is interested in a certain structure or organ, it is evident that photons emitted also from over- and under-lying activity along the projection lines will contribute to the signal recorded in a particular pixel. This generally reduces the image contrast and the detectability for small lesions and is the underlying reason for moving to 3D imaging techniques.

### 4.2. SPECT Tomographic Image Reconstruction

**Figure 2:**
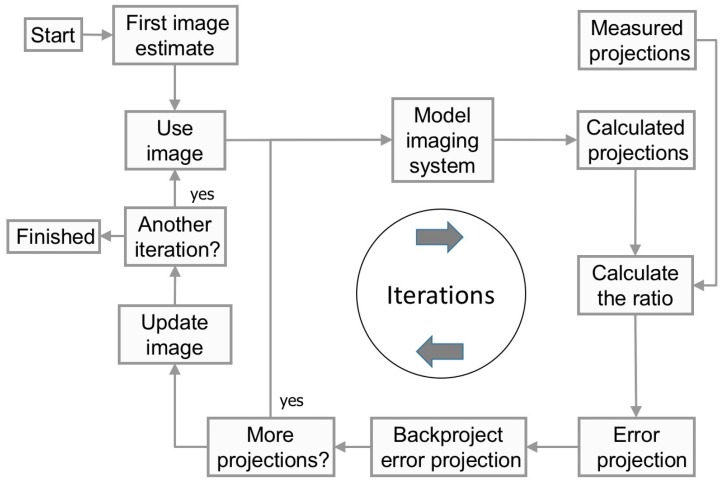
Flow-chart of the iterative reconstruction process.

The idea behind tomography is to recreate the depth information along the projection line. Since it is not possible to extract this information from a single projection, SPECT is based on acquiring multiple projection by rotating the camera around the patient and obtaining projections as a function of the projection angle. In a reconstruction, it is assumed that the activity distribution remains stationary throughout the acquisition. This means that the difference in spatial distribution of counts in the acquired projection images only is a function of the projection view. The task for any reconstruction method is then to estimate the internal distribution in the patient that would provide the acquired data. For many years, Filtered Back-Projection (FBP) was the golden standard of reconstruction methods, but today, the clinical use of iterative reconstruction methods is established. This family of reconstruction methods all include a computer model of the imaging system. Figure 2 shows the principal steps in an iterative reconstruction method. From a first estimate of the internal source distribution, the computer model calculates a projection image. This image is then compared with a measured projection to determine where in the modelled image differences occur. Most reconstructions in nuclear medicine use the Maximum-Likelihood Expectation Maximization algorithm (ML-EM) (see, for example, [7,8]) for this comparison step because of the nature of the noise in the projection being Poisson distributed. The comparison metric in the ML-EM is the ratio between the measured and modelled data, meaning that if the ratio converges to unity, then the calculated projection matches the measured one. In order to achieve convergence, the first initial estimate of the image needs to be modified. This modification can be made by back-projecting the ratios to form a tomographic error image. After all projection angles are processed, the initial estimated images of the activity distribution are multiplied by the error images, and the procedure is started again. This process is then called one iteration and, thus, means one improvement of the images. The iterations continue until the difference between the estimated and measured projections (the ratio) is below some pre-defined criteria. After a predefined number of iterations, the process is stopped, and the estimated activity distribution is regarded to reflect the internal distribution in the patient since the projections from the computer model and the real camera have reached a sufficient similarity.

**Figure 3:**
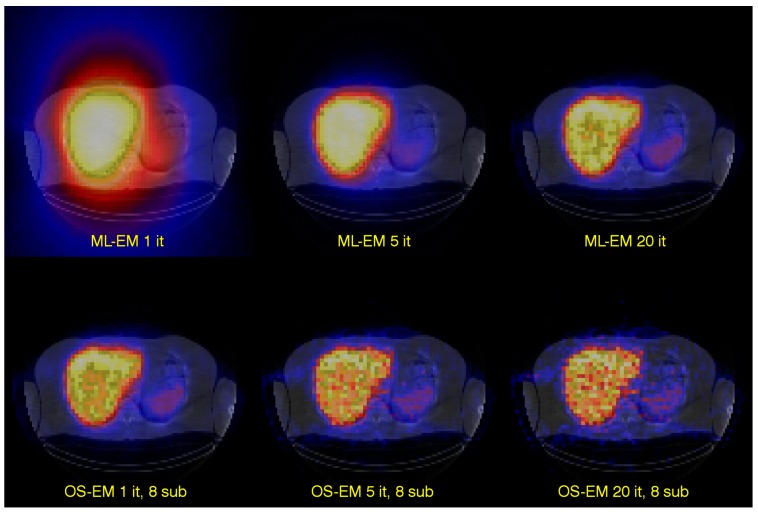
The figure shows tomographic images of the ^111^In-labeled monoclonal antibody distribution in the liver. The upper row shows images reconstructed with ML-EM and where one, five and 20 iterations have been used. The lower row shows corresponding images reconstructed with OS-EM.

A version of the ML-EM algorithm is the ordered-subsets expectation-maximization algorithm [9], which, due to the improved calculation efficiency, has made the iterative reconstruction methods practical for clinically routine studies. In many ways, the algorithm is the same as the ML-EM algorithm, but with an important difference in how the reconstructed image is updated. In ML-EM, the image is updated after having processed data from all projection angles. In OS-EM, the estimated image is updated already after only a sub-set of uniformly-distributed angles has been processed. For a 120-angle SPECT acquisition, some typical settings could be to assign about 6–10 angles per each of the subset. This means that the reconstructed images is updated about 12–20 times within each iteration. Convergence is thus reached much faster and with an accelerating factor, relative to the ML-EM algorithm, roughly equal to the number of subsets. This improvement is shown in Figure 3.

## 5. Limitations with Scintillation Camera Imaging

**Figure 4:**
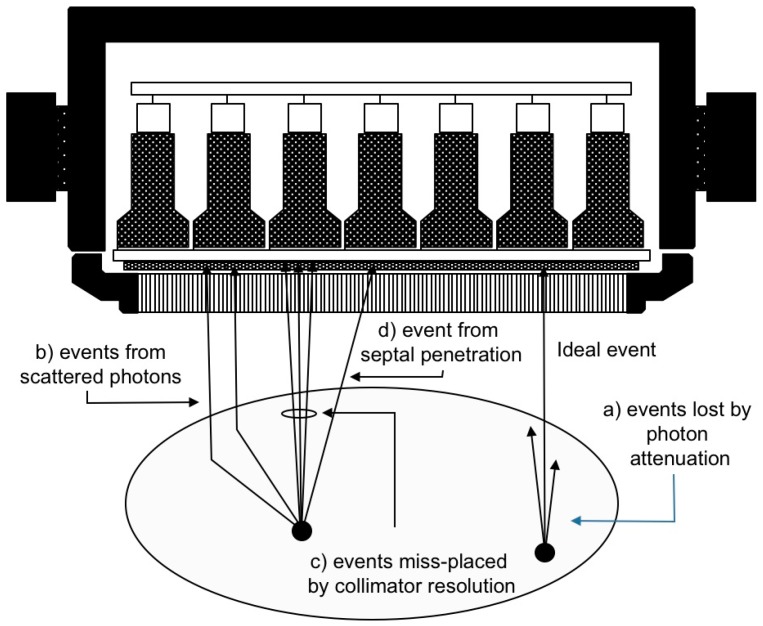
Schematic sketch showing the principles behind photon attenuation, scatter and collimator resolution effects (geometric resolution and effects from septal penetration).

There are several difficulties associated with the determination of the in vivo activity distribution in absolute units. Since the scintillation camera only registers counts (i.e., detected events), the measured values need to be calibrated in order to reflect the physical quantity activity (MBq). The relation between count rate and activity is generally not linear and constant, but a function of: (a) the absorption of photons in the patient (attenuation) that reduces the expected number of detected events; (b) the unwanted contribution of events from photons scattered in the patient; (c) the poor spatial resolution; and for some radionuclides, such as ^131^I and ^90^Y, also (d) septal penetration. Besides these major effects, other additional effects may play a significant role, such as count rate limitations in the detector system and patient respiratory movements. This holds true for both planar imaging and SPECT. The first three effects (a–c) will be discussed further in the text below. Figure 4 shows schematically the major types of physical processes that affect the count distribution.

## 6. Activity Quantification in Planar Imaging

### 6.1. Geometrical-Mean Attenuation Correction

**Figure 5:**
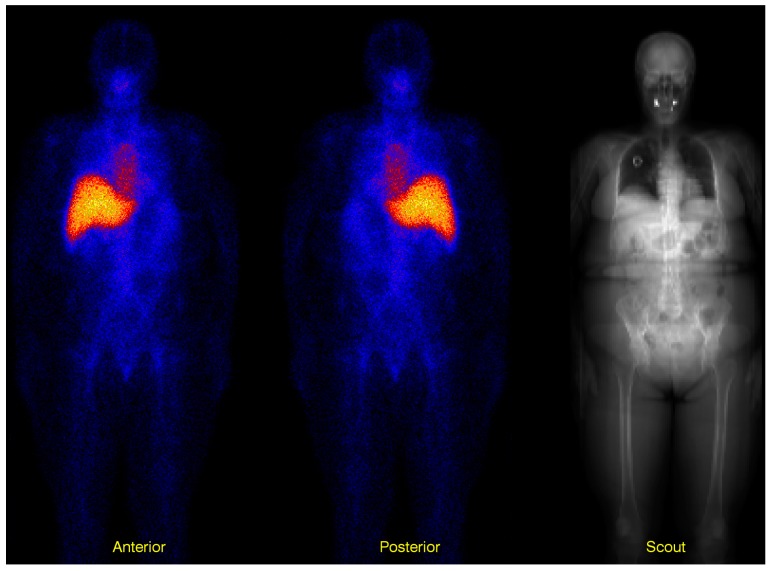
Three images showing anterior and posterior whole-body images. Before applying the geometric-mean on a pixel-by-pixel level, the posterior image is mirrored. The image to the right is the scout image whose intensity describes the amount of photon attenuation in the patient. Bright areas indicate a large probability for photon attenuation. This image is acquired with the X-ray unit, and the values are scaled to represent the attenuation factor for the relevant photon energy. The spatial misregistration between the scout image and the two planar images is accounted for by an image registration procedure, tailored for whole-body images, as described further down in the text.

The geometric-mean method [10] has been widely used for activity quantification of 2D planar imaging for dosimetry calculations. The method is based on acquiring two projections in opposite views (usually anterior and posterior as shown in Figure 5). If one considers a point source in a uniformly-attenuating media, the count rate within a region of interest (ROI) in the Anterior (AP) and the Posterior (PA) images can be described as:

(4) $$\begin{array}{l} C_{AP}=C_{o}\;e^{-\mu \left(hv,Z\right)d}\;\\ C_{PA}=C_{o\;}e^{-\mu \left(hv,Z\right)\left(T-d\right)} \end{array}$$

where *C_o_* is the count rate measured “in air”, that is without any surrounding attenuating media, μ is an average attenuation coefficient for the particular photon energy, *hv*, and tissue composition, *Z*, *T* is the total thickness of the patient at the position of the point source and *d* is the source depth in the anterior direction. Applying the geometric mean to the registered counts,$\;C_{AP}$ and $C_{PA}$, will cancel the dependence on *d*, and the effect of attenuation will become a function of *T*, following:

(5) $$\sqrt{C_{AP}\cdot C_{PA}}=\sqrt{C_{o}{}^{2}\;\cdot e^{-\mu d}\;\cdot \;e^{-\mu \left(T-d\right)}}=C_{o}\cdot \sqrt{e^{-\mu T}}$$

Equation (5) is based on the assumption of a point source, uniform attenuation and no contribution from scatter or over- and under-lying activities. For imaging with parallel-hole collimators, the registered counts in the whole camera field of view can be considered independent of the distance between the source and the camera (within reasonable distances). Therefore, the total count rate *C_o_* can be easily converted to activity by using a calibration factor, *K_air_* = *C_ref,air_*/*A_ref_*, the so-called system sensitivity, which has the unit s^−1^Bq^−1^. The factor is calculated from a calibration measurement of the radionuclide with a known activity *A_ref_* and where the count rate *C_ref_* is measured for relevant collimators and energy window settings. This implies that the source activity *A* can be determined according to:

(6) $$A=\;\frac{1}{K_{air}\;}{\left(C_{AP}\cdot C_{PA}\right)}^{1/2}\cdot e^{\mu T/2}$$

The above equation is in principle only valid for a point source. For extended sources, a correction for the self-attenuation in the source may be needed by using a correction factor [10].

### 6.2. Measured Attenuation Map

The geometric-mean method assumes an attenuation factor, $e^{\mu T/2}$, that is a function of the total thickness *T* of the patient for a particular position and an attenuation coefficient. As individual terms, these two parameters can be difficult to estimate. However, one solution is to measure the whole attenuation factor from a transmission measurement. If an external source is mounted on the opposite side of the patient relative to the scintillation camera, an image of the transmitted photons can be measured. If this image is normalized to an identical measurement, but without the patient in position, then the square root of the ratio of these two images will estimate the factor $e^{\mu T/2}$ in Equation (6). The attenuation factor can be calculated from a defined ROI or even down to a pixel-by-pixel level. The advantage of this method is also that the factor takes into account non-uniform attenuation. An image of the attenuation factor can be measured using a flood-source of ^57^Co that often is available and used for quality control purposes. However, due to the relatively low activity in such a flood source, a relatively long acquisition time needs to be used in order to maintain a reasonable signal-to-noise ratio. Furthermore, the spatial resolution of the transmission image will be determined by the scintillation camera and the particular collimator used.

In Lundadose, we have implemented an in-house-developed method for transmission measurement on SPECT/CT systems where the X-ray unit from the CT is used to obtain an image of the attenuation factor by extending the scout measurement normally used to determine the CT axial coverage, to measure the whole length of the patient [11]. An example of such a scout image is shown to the right in Figure 5. The advantages of this are several. Firstly, it is a fast acquisition in the range of 1–2 min and with a relatively small radiation dose to the patient. Secondly, the signal-to-noise ratio is high, so the scaling to the appropriate photon energy is relatively straight-forward. Thirdly, the spatial resolution is high so that the scout image can be used for other purposes than attenuation correction, such as ROI drawing. A practical problem that can occur depending on the camera system is that because the original purpose of the scout image is to provide an estimation of the axial coverage of the CT study, the camera system sometimes scales the image values into arbitrary units. The outcome of this is that scout images from thin and obese patients will have the same maximal image value. We have detailed experience from two camera systems, where for the first system, we could retrieve a scaling factor compensating for this by guidance from the vendor, using data located in a system log-file [11]. For the second system, this was not possible, and for this reason, we made a study that showed that there was a relationship between patient weight and the necessary scaling factor [12]; this is the method we currently use.

### 6.3. Scatter Correction: Planar Imaging

Scatter correction in planar imaging can be implemented by the use of an effective attenuation coefficient in the geometric-mean method [13]. It can also be made using the Triple-Energy Window method (TEW), which is based on a simple subtraction of estimates of the scatter contribution in the projection data [14]. The scatter is estimated by acquisition in additional, narrow energy windows located in close proximity to the main energy window. It is assumed that the scatter in the main energy window can be described by the average of the data obtained from the two adjacent energy windows, taking into account differences in energy window widths. The correction is implemented using the following equation,

(7) $$C_{main}^{\prime }=C_{main}-\;\left[\frac{C_{low}}{W_{low}}+\frac{C_{high}}{W_{high}}\right]\cdot \frac{W_{main}}{2}$$

where *C* is the count rate recorded in an energy window with width *W*.

**Figure 6:**
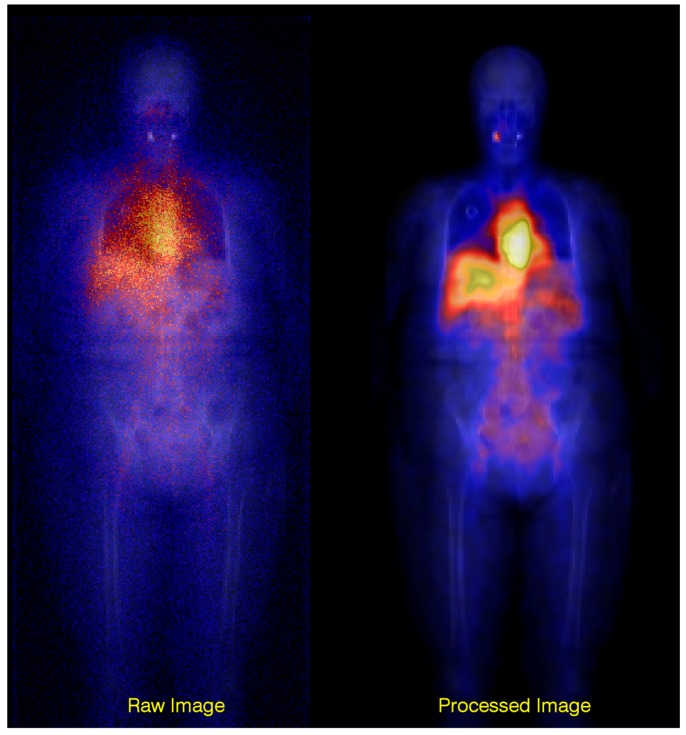
Overlay of a raw and processed planar scintillation-camera image on a scout image. The improvement in the appearance of the processed image, i.e., after attenuation and scatter corrections, is marked.

In Lundadose, scatter correction is made using a Wiener filter [15,16,17] that automatically adapts its cut-off frequency to the image-noise level. The filter uses pre-calculated scatter kernels as input and performs the devolution in the frequency domain. The scatter kernels are calculated using Monte Carlo methods for a geometry defined as a point source in a water-filled elliptical phantom. The kernels are stored as a function of source depth and are properly normalized to the total-to-primary ratio, which thus quantitatively compensates for the scatter contribution. By “primary”, it is meant the ideal events in Figure 4, i.e., events due to photons that have passed unscattered through the object and through the collimator holes. By “total”, it is meant the total counts, including primaries as well as counts originating from scatter in the object, from collimator penetration and from photons backscattered behind the scintillation crystal. The filter also has a sharpening effect on the image, owing to the deconvolution operation. A drawback with this method is that the scatter kernels only reflect one single material and do not take differences in the scattering probabilities of different tissues into account. An advantage is that the combined use of a high-resolution scout image for attenuation correction and scatter compensation makes a significant difference to the final image quality and can be used also for “difficult” radionuclide imaging situations, such as bremsstrahlung imaging of ^90^Y [18,19]. Figure 6 shows a comparison between raw and processed bremsstrahlung images using these methods.

### 6.4. Image Registration: Planar Imaging

When acquiring sequential images over several days, it is impossible to achieve exactly the same patient position at each time of measurement. Technical equipment, such as lasers and fixation, can certainly provide support, but still differences in the patient position will remain. The purpose of image registration is to transform the sequence of images geometrically so that in the end, the pixel values in each of the images are associated with the same position in the patient. In Lundadose, co-registration of whole-body planar images is included for several purposes. Firstly, the scout image is only acquired on one occasion, while the whole-body images are acquired on several occasions with patient re-positioning as a consequence. In order to perform attenuation correction on a pixel basis, each of the whole-body images thus needs to be registered to the scout image. Secondly, one single set of ROIs can be used for organ delineation, which is an advantage in view of the time required for the evaluation of time activity curves and absorbed dose calculation. Thirdly, in some cases, the scout image can be used to draw ROI in regions where it is difficult to define the boundary in the WB images, such as the lung border, e.g., the image registration is accomplished by automatic optimization of a spatial transformation, especially constructed for whole-body planar images, with regards to the mutual information between images [20,21,22]. In effect, the spatial deformation is a combination of global and local transformations of regions roughly covering the head, torso, left leg and right leg and includes translation, rotation, shearing and second degree curving [23].

### 6.5. Background and Overlapping Activity

Planar images suffer from a lack of resolution along the depth dimension, and the counts detected in an ROI will inevitably originate from activity in the tissue of interest, as well as over- and under-lying structures. To some extent, this can be compensated for by delineating background ROIs adjacent to the organ ROI to estimate the activity in the general background. The contribution from the background into the organ ROI is then subtracted, where the background ROI counts are first rescaled to take the different ROI sizes, i.e., the number of pixels, into account. In Lundadose, the correction for background activity and overlapping activities is made on a pixel basis in the quantified activity images [15]. In addition to drawing adjacent ROIs, the different body thicknesses over the organ and background ROIs are taken into account. These thicknesses are estimated from the attenuation map, which represents the total attenuation, i.e., the integral of attenuation coefficients over the total patient thickness at different positions. Division by the linear attenuation coefficient for water thus yields an image that represents the distribution of water-equivalent thickness over the patient. For background correction of the organ ROI counts, the activity is assumed located in either of two different compartments, organ or background, whose respective volumes are defined by the ROI area multiplied by the respective compartment thickness. These thicknesses are calculated as the product of the total patient thickness at the ROI location and pre-calculated fractions, as derived from the MIRD and Zubal phantoms [24,25]. For instance, for the liver, the fraction of the total phantom thickness was determined as 0.61 for the Zubal phantom and 0.58 for the MIRD phantom [15]. In a patient, the total-body thickness over the liver is determined from the scout-derived water-equivalent thickness image. The liver thickness is then calculated by multiplication to the assumed liver fraction of 0.6, whereas the background-compartment thickness is calculated from the fraction 0.4. The activity in the background compartment is determined as the product of its volume and a pseudo background-activity concentration, as estimated from the background ROI. This is placed in a location where all counts originate from “background”, and the volume is calculated using the background-compartment thickness-fraction of one. A correction for the mean source extension [26] is also applied on the background-corrected ROI values.

## 7. Activity Quantification from SPECT Imaging

**Figure 7:**
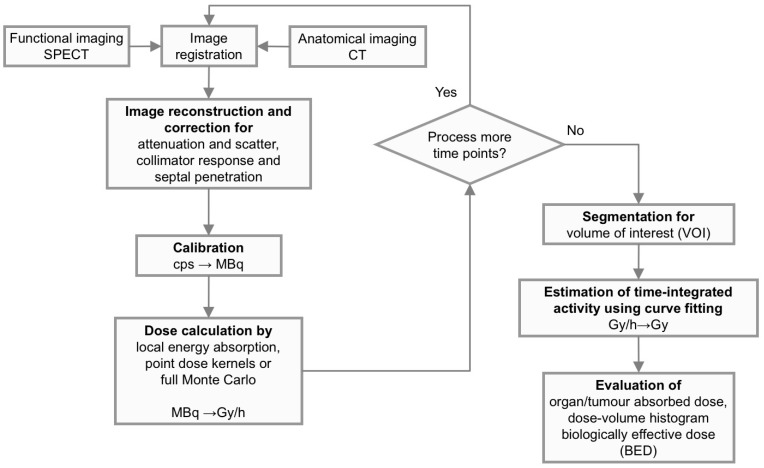
A flow-chart describing the essential steps needed to obtain quantitative 3D activity and related absorbed-dose images. If using a combined SPECT/CT system, explicit registration of SPECT and CT images is not required, since positioning and interpolation are inherent in the imaging system. In most commercial SPECT/CT systems, necessary corrections are embedded within the iterative reconstruction method, at least for non-homogeneous attenuation correction, scatter correction and compensation for collimator resolution. For most radionuclides used in radionuclide therapy, the path-lengths of the electrons (i.e., the particles that deliver the energy) are short compared to the voxel size, and therefore, a simple scaling, by assuming a local energy deposition within a voxel, will be justified. This makes 3D dosimetry based on SPECT/CT relatively straight-forward.

The schematic flow-chart shown in Figure 7 describes the most important parts in the procedure to obtain quantitative 3D activity images and to further steps necessary to process in order to estimate the absorbed dose to the target volume. Note that in many cases, the steps are also applicable to planar images, as has been described above.

### 7.1. SPECT/CT Acquisition

The setup of the acquisition protocol is important because all images for dosimetry and visual evaluation rely on the good quality of acquired data. However, several constraints need to be considered. Firstly, the total acquisition time is limited by the time a patient can stay comfortably on the couch. For SPECT, a sufficient number of projections are required to avoid reconstruction artefacts due to improper angular sampling. For a given overall acquisition time, more projection means a shorter time for acquisition per projection. The thickness of the scintillation-camera crystal has importance, since it affects the number of photons that pass through without interaction and thus may decrease the count rate for high-energy photons. This can partly be compensated for by a thicker scintillation crystal, but ME and HE collimators are then required, which have less sensitivity compared to low-energy collimators. HE collimators also have thicker collimator septa and hole diameters, which affect the spatial resolution. When imaging patients during ongoing therapy with high administered activities, the camera performance regarding the count rate is important. If large count losses occur due to dead time, etc., the scaling from counts to activity by the calibration factor will underestimate the activity and thereby the absorbed dose.

### 7.2. Image Reconstruction and Activity Quantification

Image reconstruction and activity quantification are closely related to each other when using iterative reconstruction methods. Attenuation correction is in Lundadose made by using an attenuation map derived from a CT study. Calibration from Hounsfield units to density values is made by using a bi-linear expression obtained from a calibration for the current CT unit. The voxel-based density values are then multiplied by the tabulated mass-attenuation coefficients relevant for the current photon energy. Scatter correction is made using the ESSE (Effective Scatter Source Estimate) method where pre-calculated Monte Carlo-based scatter kernels are used to estimate the scatter in the photo-peak window [27]. Kernels are calculated for the relevant energy window settings, camera energy resolution and photon energies using the SIMIND (Simulating Medical Imaging Nuclear Detectors) Monte Carlo code [28]. Collimator response compensation can also be performed using either a geometrical Gaussian estimate of the point-spread function or from pre-calculated Monte-Carlo-based kernels in which also effects from septal penetration, collimator scatter and scatter originating from materials behind the crystal can be included. The software for quantitative SPECT reconstruction has been developed by Eric Charles Frey and colleagues at the Johns Hopkins Medical Center, Baltimore, MD, USA.

### 7.3. Image Registration: SPECT

Although combined SPECT/CT systems provide inherently co-registered CT and SPECT images, image registration may be required in dosimetry applications due to the requirement of imaging at multiple time points. The spatial complexity and detail included in the spatial transformations are largely determined by the application and may include rigid, affine or non-rigid methods. For voxel-based dosimetry calculations, accurate registration of the time series of images may be central to achieve accurate results in the estimate of the time-integrated activity. In Lundadose, we have implemented a non-rigid method, based on a B-spline free-form deformation, and using the mutual information as a metric of similarity [29,30]. Since the level of anatomical detail is higher in CT images, registration of serial SPECT/CT images is accomplished using CT-CT image registration for determination of the spatial transformations, which are then applied also to SPECT images. A requirement is then to pay attention to proper normalization of image values during resampling and interpolation, to ascertain that the quantitative image values are not deteriorated by the image registration. The method implemented in Lundadose was successfully evaluated in a clinical study involving acquisition of 6–7 SPECT/CT images over one week and covered anatomical regions from the head-and-neck region down to the pelvis [29].

## 8. Absorbed Dose-Rate Calculations

### 8.1. Planar Imaging

Since planar imaging does not resolve the volume of the organ, absorbed-dose rate calculations have to rely on pre-calculated S values obtained from computer phantoms of the patient. Often, tabulated S values are used according to the formalism from the Medical Internal Radiation Dose (MIRD) committee [31]. The activity, obtained from quantitative calculations, is multiplied with these S values. The limitations of this method are several. Firstly, the absorbed-dose rate is calculated from a geometric description of a model patient with a usually homogeneous distribution inside each of the organs. The difference in organ masses between the real patient and the phantom can be relevant and can be taken into account, although differences in shapes cannot be accounted for.

### 8.2. SPECT Imaging

In SPECT, absorbed-dose rate calculations are made by dividing the values in the images of the rate of absorbed energy with the matching density images obtained from the CT study, taking also into consideration the voxel dimensions. Then, each voxel value itself represents the absorbed dose rate (J/kg/h) in the location corresponding to the voxel location in the patient. Organ absorbed-dose rates can be obtained by segmentation of the absorbed-dose rate images and from these, calculate the average. Essentially three different methods to calculate absorbed energy images can be used, including: (a) assuming local energy deposition; (b) point-dose kernel convolution; and (c) a full Monte Carlo radiation transport calculation.

#### 8.2.1. Local-Energy Deposition

This method is the simplest, but is also limited in accuracy. It is useful for situations where the range of the charged particles that impart energy to the tissue is lower than the spatial resolution of the SPECT-derived activity image and its voxel size. For most charge-particle emitters used in radionuclide therapy, this assumption is valid. Even for ^90^Y with a maximum track-length of about 11 mm, most of the emitted particles do not exceed a projected path-length of about 4–5 mm (i.e., a measure of the radial distance from the emission point), a distance that is comparable with a voxel dimension. This means that most of the kinetic energy from the charged particles will be deposited within the same voxel as from where it was emitted. For photons, however, the path-length is generally longer than a voxel dimension, so in this case, the assumption of local energy deposition will not be valid.

#### 8.2.2. Point-Dose Kernel Convolution

This method is a convolution-based method to estimate the spread of energy from a point source. The implementation is in principle similar to low-pass filters used in image processing to smooth images and reduce noise. The method assumes that decays occur in a voxel, and then from Monte Carlo simulated point-dose distributions, the distribution of energy depositions in nearby voxels can be estimated. Point-dose kernels have been calculated and tabulated for mono-energetic photons and electrons, beta-particles and complete decay schemes [32,33]. Lately, so-called voxel S values have been published that are calculated for certain matrix and voxel size combinations for an easy implementation [34]. The drawback with point-dose kernels is that they are determined for a single material (often water), and thus, do not account for tissue and density heterogeneities. Currently, the Lundadose methods do not use this approach for absorbed dose calculations.

#### 8.2.3. Full Monte-Carlo Particle Transport

The most accurate, but also the most time-consuming method for absorbed dose rate calculation is to use a full Monte Carlo-based transport program. These programs use quantitative SPECT images and registered density images to model the radiation transport. The programs generally sample decay locations from the quantitative SPECT images by assuming that each voxel value reflects the activity in the particular location in the patient that corresponds to the voxel location. The transport of the emitted particles (photons and electrons) is then simulated in the CT-derived density image volume, and the energy depositions at each interaction site are scored in a separate energy matrix that has equal size and voxel dimensions as the SPECT image set. The result will then be a set of absorbed-energy rate images.

Lundadose uses an in-house written EGS4 program [35] for the radiation transport simulation. This program [36] is written in a language called Mortran. Although the EGS4 program package has in many applications been replaced by the more updated EGSnrc program package [37], our program calculates accurate absorbed doses for the voxel dimension used in clinical applications. In our clinical patient studies, we assume local absorption of kinetic energy from the charge-particles and only follow the photons by a full Monte Carlo simulation in order to estimate the cross-doses between organs. This is made based on the fact that the SPECT images that are used as input have a spatial resolution that is larger or comparable with the projected range of the electrons [38]. This means that the SPECT images already from the reconstruction step are blurred. An electron transport calculation would essentially imply a second convolution process, where the first then relates to the blurring induced by limited spatial resolution. This is important because if the deposited energy is falsely redistributed over too many voxels, then the absorbed-dose rate in each voxel will be underestimated.

## 9. Time Activity Curves

The pattern of the accumulation and excretion of radiopharmaceuticals is governed by passive physiological mechanisms, as well as active mechanisms, such as receptors. The amount and rates of radiopharmaceutical uptake and excretion varies amongst individuals. These parameters have a direct bearing on the absorbed dose, and for an individualized treatment, they need to be determined for each patient. The method used to determine so-called Time-Activity Curves (TACs) for different organs and tissues is to perform imaging at several time points after administration, for instance during the first week, and to determine the activity concentration in different tissues at each time. As the absorbed dose is associated with the area under this TAC, the simplest analysis method is to apply trapezoidal integration up to the last time point and then make an assumption, for instance based on the radionuclide physical half-life, for extrapolation beyond the last data point. A somewhat more sophisticated method is to fit a predetermined model curve to patient data. Most commonly-used curve shapes are sums of exponential curves with different rates, where the number of terms is generally limited by the number of data points that are available. Even more sophistication can be incorporated into this analysis, for instance by taking more than one organ into consideration at a time, using compartmental modelling [39]. A systematic testing of different curve shapes can also be applied by performing fits to different curve shapes and determining which model fits the data best using a statistically-based criterion, such as the Akaike or F-test, e.g., [40]. Attempts have also been made to compile patient data in order to use the group behavior as a prior when applying Bayesian techniques. Lundadose includes different options for TAC fitting and for choosing automatically between them based on the Akaike and F-test. However, we generally find that, given the limited amount of data points for each patient, the usefulness of such automatic procedures is not obvious, and in patient studies, we generally use one fixed model, which is fitted to each patient. The initial estimates for the TAC fitting are of importance and are determined based on data and the radionuclide physical half-life. For instance, a first, approximate determination of the rate of accumulation is determined as the linear slope between the first data point and the point where the activity is at maximum, while the rate of excretion is initially estimated as half the radionuclide half-life. From the fitted model, the area under the TAC is determined by analytical integration.

## 10. Absorbed Dose Calculations

### 10.1. Planar Imaging

The general way of calculating the absorbed dose from planar images is to use organ-based S values (Equation (3)) multiplied by the time-integrated activity obtained from the TAC. Of the more commonly-used S values are those derived from the Cristy-Eckermann phantom, also included in the Olinda software [41] and available on-line [42]. For patient-specific dosimetry, these phantoms should be used with caution because these are developed for the purpose of risk estimates of stochastic effects associated with diagnostic nuclear medicine examinations. Such risks are estimated for populations and not individuals, and the phantom is designed to represent a reference of an ‘average’ male, female or young person. Nevertheless, if these S values are used, some tailoring can be made to an individual patient by, for example, scaling the S values to the individual organ masses.

### 10.2. SPECT Imaging

Absorbed doses can be calculated by registering multiple quantitative SPECT studies so that each VOI is aligned throughout the image sets. TACs can then be generated and absorbed doses calculated in principle down to the voxel level. However, as discussed earlier, the voxel dimensions are always smaller than the spatial resolution of the SPECT imaging system, so the significance of individual voxel values is probably low. Furthermore, due to statistical reasons, related to image noise and potential reconstruction artefacts, voxel-based curve fitting can be unstable and may result in odd local TACs if no boundary conditions are applied. In a paper published by our group related to dosimetry and pre-therapy dosimetry of ^90^Y/^111^In-labelled monoclonal antibodies, we used this technique, but with a trapezoidal calculation instead of a curve-fitting procedure [19]. The application was here the estimation of the absorbed dose to the liver, and because of the large organ and, as a consequence, many voxels, we found the method to be reliable. It should be noted that the number of SPECT/CT time-points in that study were seven for the ^111^In pre-therapy study and six for the ^90^Y therapy study.

### 10.3. SPECT/Planar Hybrid Method

This method is a combination between planar and SPECT imaging [43]. In short, the idea is that the shape of the TAC is determined from multiple planar images while the amplitude of the TAC is determined by normalization to the activity determined from a quantitative SPECT image acquired at one time point. The normalization factor is determined as the ratio of the activities obtained from planar and SPECT studies on the same time point, and the same ratio is then used for all time points. This method is a compromise and is useful when it is not possible to perform multiple SPECT/CT studies due to time constraints or if the whole-body information is of higher priority. The shape of the TAC is still affected by the limited quantitative accuracy for planar imaging, but the method can improve the dosimetry when compared to using data from planar imaging only.

## 11. Biologically Effective Dose

The Biologically Effective Dose (BED) is a concept within the framework of the Linear-Quadratic (LQ) radiobiological model. It relies on the idea of equi-effective treatments, i.e., treatments that produce the same probability of inducing a specific clinical (biological) endpoint. The main use of BED is probably in EBRT, where it is a clinically-accepted method for conversion between different fractionation schemes. In RNT, it has been applied to take different fraction absorbed doses and absorbed-dose rates into account when correlating to the biologic effect [3,4]. The BED is defined as the absorbed dose that would be required to induce a given biologic effect, if the dose were delivered at infinitesimally small fractions or low absorbed-dose rate. The so-called Lea–Catcheside factor is a function incorporated in the expression for the BED to take the effects of a variable dose-rate into account. The value of this function for a particular irradiation situation depends on the rate of repair and the absorbed-dose rate, which in turn depends on the effective half-life of the radiopharmaceutical in the organ being evaluated. Within Lundadose, we have developed a general method for calculating the BED, which is based on convolution between the functions describing the absorbed-dose rate and the repair [44,45]. This method has the advantage of being flexible in terms of different absorbed dose-rate patterns and also allows for more complicated repair functions compared to the most commonly-applied mono-exponential repair model.

## 12. Limitations with Image-Based Dosimetry

One of the potential advantages with image-based dosimetry on the voxel level is its ability to provide absorbed dose distributions reflecting much smaller volumes than whole organs. This means that, in theory, it is possible to determine the variation in absorbed doses down to a level defined by the voxel dimensions. This information can then be used to describe the variation of absorbed doses together with maximum, minimum and mean values, possibly in combination with a dose-volume histogram. However, when using 3D SPECT images as input for absorbed dose calculations, caution and conservatism must be maintained. It is well known that many parts in the imaging process and the reconstruction methods have a significant impact on the distribution of image values and, thus, the voxel values of the absorbed dose. The values in individual voxel variation may thus be caused by other reasons than an actual variation in the absorbed dose, and quite possibly, there is a smallest volume that can be considered adequate for quantification, in view of these limitations.

### 12.1. Spatial Resolution

The reconstructed spatial resolution is about 10–15 mm for an ME or HE collimator. For example, even if the dimension of a spherical source is on a sub-millimeter level, the apparent size of the sphere in the image will be displayed as a diameter close to the spatial resolution. This means that the input data for image-based absorbed dose calculations is from the beginning already to some extent false whether or not one uses the assumption of local energy deposition, point-dose kernel convolution methods or a full Monte Carlo calculation procedure.

### 12.2. Image Noise Related to Reconstruction Parameters

One of the properties with iterative reconstruction methods is that the image noise tends to increase as the number of iterations increases. On the other hand, small objects need a higher number of iterations before convergence is achieved. This means that optimization is needed, in view of the purpose of the image. A smaller number of iterations produces smoother images, but small objects are then difficult to visualize properly. Higher iterations can generate considerable noise variations, but the average value in a VOI may be close to the correct value. Often, smoothing post-filtering methods are applied after reconstruction to decrease the variation and make the images more visually appealing, but such low-pass filtering also spreads out the signal (i.e., the absorbed energy over a larger number of voxels), leading to generally smaller voxel absorbed doses since a larger number of voxels share the value of the deposited energy.

### 12.3. Reconstruction Artefacts

When applying compensation for collimator resolution, artefacts can appear that result in a ring-shaped increase or decrease of the count level [46]. A collimator compensation works in such a way that, from the PSF (Point-Spread Function), i.e., the system response when measuring a point-source, frequencies that describe details are recovered, which however, may be absent in the projection images due to the limited spatial resolution. As a result, significant under- and over-corrections of image data may occur during the compensation process that can cause a wave-formed pattern in the image. These are often seen as intense borders around organ activities or as a cold part of a spherically-shaped source, which without collimator compensation would become a uniform intensity [46].

### 12.4. Example of Dose-Volume Histograms

**Figure 8:**
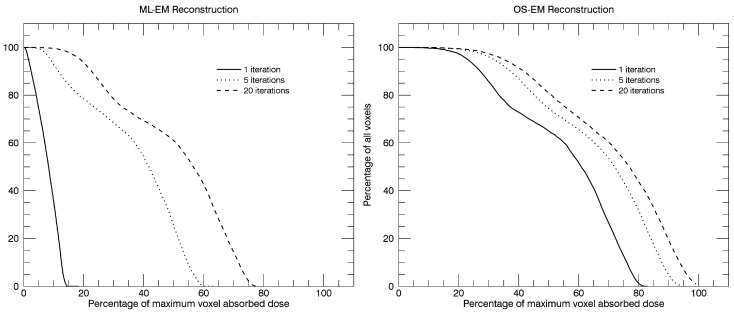
Calculated integral dose-volume histograms (DVHs) obtained in a VOI defined over the whole liver region for the six reconstructions as shown in Figure 3.

The frequency of voxel values within the liver for the six reconstructions shown in Figure 3 have been calculated, and from these distributions, integral dose-rate volume histograms (VHs) have been generated and are shown in Figure 8. The curves display the percentage of the total number of voxels that have an absorbed dose-rate value that is lower than or equal to a certain percentage of the maximum dose rate, as obtained for the highest number of iterations in the OS-EM reconstructed image. Since the purpose of the graphs is to show an example of the variation due to the reconstruction method and the number of iterations, no compensations have been applied for photon attenuation, scatter and collimator response. Local energy deposition is assumed.

## 13. Clinical Example

Lutetium (^177^Lu) is today a radionuclide that is being used widely for targeted radionuclide therapy due to its favorable decay properties for therapy (β-particles for therapy and two relatively low-energy photon emissions that are useful for imaging) and its reliable biochemistry to biomolecules used for tumor targeting. Today, the most used clinical application of ^177^Lu treatments is probably in peptide receptor radionuclide therapy (PRRT) and where ^177^Lu is attached to DOTA^0^-Tyr^3^-octreotate (^177^Lu-Dotatate) [47,48]. In the majority of cancer centers, patients are administered ^177^Lu-Dotatate with a fixed activity amount of 7.4 GBq, and such administrations are given at four occasions with an interval of approximately two months. The principal organs at risk in this therapy are the kidneys and bone marrow. Since kidneys are late-responding tissues, i.e., the potential damage from deterministic tissue reactions may be clinically detectable first after one or several years after treatment, it is of concern to follow the individual renal absorbed dose, as well as the kidney function. The renal absorbed dose that can be tolerated in this treatment is yet not known, but there is hope that this knowledge will improve following detailed dosimetric studies [2]. For instance, in a preliminary study at our institution and using limiting renal BED values described below, we found that 79% of the patients could have received more than the normally used four treatment cycles (abstract OP292 in [49]).

As an example of such personalized dosimetry, we will here describe the procedure that we currently are working with at our department at Lund University Hospital and in collaboration with the Sahlgrenska University Hospital, Gothenburg Sweden, using the Lundadose dosimetry tools. This is an ongoing clinical trial of a treatment procedure based on administered activities of 7.4 GBq and where new fractions are given until the calculated total BED dose of the kidneys has reached either 27 Gy or 40 Gy. The selection of the two levels depends on the review of additional risk factors for the patients [47], as well as extrapolation of data from EBRT studies. Personalized image-based dosimetry is performed during every treatment cycle by using both planar and SPECT scintillation camera imaging. Ideally, we would prefer to perform SPECT/CT on all time-points to avoid the known limitations of planar activity quantification. However, because of the limited axial FOV of the SPECT camera (40 cm), that would demand acquisition in multiple bed positions, and together with a need for planar Whole-Body (WB) images in order to assess the tumor burden, we use the compromise of the hybrid SPECT/planar dosimetry method. WB images are acquired at four time points in the intervals of 0, 24 or 48, 96 and 168 h post-administration. The WB images are quantified for activity using the geometric-mean method and patient-specific attenuation correction using a measured X-ray scout image (Section 6.2). Scatter correction is made with the Wiener filtering method (Section 6.3), and here, we calculate the ^177^Lu scatter kernels with our Monte Carlo code SIMIND. ^177^Lu emits two major photons of the energies of 208 keV and 113 keV and with an abundance of 10.4% and 6.2%, respectively. We only use the upper photon energy for acquisition in order to avoid potential problems with the down-scatter of events from the 208-keV photon into a 113-keV energy window. Due to the relatively high activity, the count-rate in the upper energy window of 15% is sufficient to achieve images of adequate count rate for quantification. The renal TACs are determined from delineated ROIs in the registered planar WB images, which each are co-registered to the attenuation map (Section 6.4) and are corrected for overlapping tissue activities (Section 6.5) [43,47].

The SPECT/CT scan is acquired at 24 h post-injection. We use 60 projections, acquiring in a 360-degree rotation mode and with a time per projection of 45 s. The SPECT system is equipped with ME collimators, and projection data are stored as 128 × 128 matrices. Tomographic reconstruction is performed by the iterative OS-EM algorithm (Section 7) using eight iterations and ten subsets, including corrections for non-homogeneous attenuation, contribution from scatter and collimator response.

To convert the counts in the reconstructed images to voxel activity, we derive the calibration factor (*K_air_* in Equation (6)) by a planar image acquisition. A solution of ^177^Lu with a known activity is filled in a Petri dish and is placed 10 cm from the camera collimator face and without any scattering material in between, so that *K_air_* will reflect the camera system sensitivity in “air”. The factor *K_air_* is calculated by enclosing the disk in the image by a circular ROI and normalizing the sum of the ROI counts to the known ^77^Lu activity and the acquisition time. The radius of this ROI is adjusted automatically so that it precisely encompasses the spill-out region, and also, background ROIs are drawn and any background counts subtracted. The use of the planar-derived calibration factor acquired in air can be understood as a calibration of the counts in the projections (which are indeed planar images). Since our tomographic reconstruction method includes accurate corrections for attenuation, scatter and collimator response, what remains in the projections after corrections are the counts as if they were generated by an activity distribution placed in air. Thus, since the reconstruction is properly normalized and takes the number of acquisition angles and time per projection into account, this simple calibration factor can be used for conversion from counts to activity also in the reconstructed image.

We calculate the absorbed-dose rate from the quantitative SPECT image with the EGS4 Monte Carlo-based program where a 3D density distribution of the patient is used and obtained from the CT (Section 8.2.3) [29]. Only energy depositions from photon interactions are included in the EGS4 computation, and the kinetic energy from electrons and β-particles, emitted from the radionuclide, is calculated using the local-energy deposition method (Section 8.2.1). VOIs are manually defined over the cortex and the medulla of both kidneys by delineation from the CT images. The spill-out of counts from a VOI, caused by the differences in spatial resolution between the SPECT and the CT images that are used for delineation, is compensated for by multiplying with a recovery factor. The value of the factor was determined by evaluation of Monte Carlo simulated images [28] of kidney uptakes in an anthropomorphic computer phantom [50] with a realistic ^177^Lu-Dotatate activity distribution [51]. From a VOI delineation, performed by the technologists involved in patient treatments, the recovery coefficient was determined. A single recovery factor is used since the kidney shape in transverse slices is relatively stable among the patients, although the kidney axial extension varies, giving different volumes. The TACs, obtained from WB image analysis, are rescaled by the ratio of the SPECT-derived absorbed-dose rate and the activity from a ROI in the WB image at 24 h. After scaling, the mean absorbed doses within the VOIs are calculated by curve fitting and analytic integration of the absorbed-dose rate curve. The BED is also calculated from the absorbed-dose rate curve [3].

## Abbreviations

The following abbreviations are used in this manuscript:

SPECT	Single Photon Emission Computed Tomography
CT	Computed Tomography
RNT	Radionuclide Therapy
XRT	External Radiation Therapy
FOV	Field Of View
BED	Biological Effective Dose
ROI	Region Of Interest
VOI	Volume Of Interest
DVH	Dose Volume Histogram
ME	Medium Energy
HE	High Energy
EGS	Electron Gamma Shower
OS-EM	Ordered Subset Expectation Maximization
ML-EM	Maximum Likelihood Expectation Maximization
TAC	Time Activity Curve
WB	Whole-Body
MIRD	Medical Internal Radiation Dosimetry
